# Health research in the Syrian conflict: opportunities for equitable and multidisciplinary collaboration

**DOI:** 10.1093/pubmed/fdab160

**Published:** 2021-05-21

**Authors:** Abdulkarim Ekzayez, Amina Olabi, Yazan Douedari, Kristen Meagher, Gemma Bowsher, Bashar Farhat, Preeti Patel

**Affiliations:** Research for Health System Strengthening in northern Syria (R4HSSS), Research for Health in Conflict in the Middle East and North Africa (R4HC-MENA), and the Conflict and Health Research Group (CHRG), King’s College London, WC2R 2LS, UK; Syria Public Health Network, UK; Union for Medical and Relief Organisations (UOSSM), UK/Turkey; London School of Hygiene and Tropical Medicine, Department of Global Health, London WC1H 9SH, UK; Syria Research Group (SyRG), co-hosted by the London School of Hygiene and Tropical Medicine, WC1E 7HT, UK; Saw Swee Hock School of Public Health, 117549, Singapore; Research for Health System Strengthening in northern Syria (R4HSSS), Research for Health in Conflict in the Middle East and North Africa (R4HC-MENA), and the Conflict and Health Research Group (CHRG), King’s College London, WC2R 2LS, UK; Research for Health System Strengthening in northern Syria (R4HSSS), Research for Health in Conflict in the Middle East and North Africa (R4HC-MENA), and the Conflict and Health Research Group (CHRG), King’s College London, WC2R 2LS, UK; Union for Medical and Relief Organisations (UOSSM), UK/Turkey; Research for Health System Strengthening in northern Syria (R4HSSS), Research for Health in Conflict in the Middle East and North Africa (R4HC-MENA), and the Conflict and Health Research Group (CHRG), King’s College London, WC2R 2LS, UK

**Keywords:** action research, public health, research

## Abstract

There is considerable global momentum from Syrian researchers, policy makers and diaspora to address health, security and development challenges posed by almost a decade of armed conflict and complex geopolitics that has resulted in different areas of political control. However, research funders have been so far reluctant to invest in large-scale research programmes in severely conflict-affected areas such as northern Syria. This paper presents examples of collaborations and programmes that could change this through equitable partnerships between academic and operational humanitarian organizations involving local Syrian researchers—a tremendous way forward to capitalize and accelerate this global momentum.

Several academic and humanitarian organizations have initiated collaborations to build new networks and partnerships for better research and policy engagement in Syria. The networks conducted two consecutive annual conferences in 2019 and 2020. Key messages from these conference include: (1) equitable partnerships between organizations and individual researchers must form the basis of conducting better research; (2) ensuring the inclusion of local Syrian researchers is crucial in the development of any viable partnership; (3) capacity strengthening in health research is urgently needed in Syria’s current phase of active conflict to inform, develop and implement strengthened and sustainable health systems in the post-conflict phase.

## Introduction

The Syrian conflict continues to devastate health systems, infrastructure and personnel, resulting in the rapidly deteriorating mental and physical healthcare needs of individuals. Syrians continue to be by far the largest forcibly displaced population worldwide, with 13.2 million in need of urgent humanitarian assistance, including more than 6 million internally displaced people.^1^^,^^2^ In a country that suffered almost 10 years of conflict, the impact on human suffering is palpable and stipulates the necessity for robust systems of evidence-based research into healthcare practice and needs. Despite the dire and urgent health needs of the Syrian population, the bulk of health research provides a very limited understanding of the systemic health needs arising in the Syrian context, focusing instead on specific but disparate health outcomes and services. There is also a vast amount of health research that focuses on Syrian refugee populations, particularly in neighbouring and European countries, but a more limited programme of research centring on the residual health sector and the remaining resident population, including Internal Displaced Persons (IDPs) which is considered to be among the most vulnerable groups inside Syria. Current health research literature on Syria seems to have very limited inclusion of institutions and researchers who remain in Syria, which raises questions on research capacity as engagement, as well as acknowledging contributions from Syria-based researchers through authorship, if any.^3^ Multiple systemic issues arise in the Syrian context requiring structured health systems research, which include, although are not limited to, the effect of health professional exodus on service delivery, the manner in which parallel health services deliver targeted outcomes across varied complex political geographies, the strengthening of health services and systems facing co-morbidities (or multi-morbidities) of physical and mental trauma alongside communicable and non-communicable disease (NCD) pressures, the education and the training of present and future health system leaders, and the application of the humanitarian development nexus in such a chronic and complex conflict.^3–5^

The health humanitarian response in Syria has been incredibly complicated yet innovative considering the challenges of cross-border aid, remote management and daily attacks on health infrastructure and personnel. It is vital to document good practices and innovative approaches through operational research. It is important also to assess the effectiveness of the humanitarian health interventions, especially those that were introduced as new interventions that were not part of the health system in Syria before the crisis. For example, the programme of Community Health Workers (CHWs) is a new approach that was introduced by the humanitarian response which has not yet been assessed in relation to quality and effectiveness. Similarly, the use of mobile health clinics targeting displaced populations on the move inside Syria has been described as an innovative and flexible approach to address the health needs, even partially, of internally displaced populations inside Syria. New designs of embedded operational research will bolster the value of health research in Syria in relation to documenting good practices and measuring effectiveness of key health interventions.

## Building effective research networks and new platforms for health research in Syria

Aiming to fill key gaps in health research and improve policies on health interventions and health system strengthening in Syria, several academic and humanitarian organizations have initiated collaborations to build new networks and partnerships for better research and policy engagement in Syria. These growing collaborations are based on the experience of the individual institutions involved in these collaborations, as well as the experience and connections of Syrian researchers, many of whom represent Syrian health diaspora in these institutions. These diverse groups include the Conflict and Health Research Group (CHRG) at King’s College London, The Syria Research Group (SyRG) co-hosted by the London School of Hygiene and Tropical Medicine (LSHTM) and Saw Swee Hock School of Public Health, the Union of Medical and Relief Organizations (UOSSM), the Syrian American Medical Society (SAMS), the Centre on Global Health Security at Chatham House and the Syria Public Health Network (SPHN). Building on the experience of the CHRG’s project, Research for Health in Conflict in the Middle East and North Africa (R4HC-MENA), researchers at the CHRG started reaching out to individual and institutional researchers in Syria to explore collaborations aimed at research capacity strengthening and building a field-informed research agenda. This was met with a similar proactive approach from UOSSM, which established a dedicated organization (MIDMAR) in Turkey for research and development. Similarly, SyRG has considerable experience engaging with field researchers in Syria specifically on health system governance and community engagement research.

Building on these collective experiences, a group of Syrian researchers based within these institutions initiated the idea of an annual international conference to provide a platform for individual and institutional health researchers to present their research interests and findings. The aim was to (1) empower researchers and entities who are Syria-based, (2) encourage more research networks and partnerships among local and international academic and humanitarian actors, (3) draw attention to the importance of health research in the Syrian context in improving the current humanitarian response as well as in shaping the future health system in Syria and (4) contribute to creating a field-informed research agenda for health in Syria. We conducted two consecutive conferences so far, and next we summarize key themes from both conferences. We then highlight the need for representing more Syrian researchers based in the country and the need for more equitable research partnerships to accompany the growing global momentum to fund large scale research and policy programmes in areas affected by chronic armed conflict and humanitarian crises.

## The First International Syria Health Research Conference—10 October 2019

Held at King’s College London in the River Room, overlooking London’s River Thames, the first international conference attracted attention from many Syrian researchers living in Syria and the diaspora across Europe, the Middle East, Asia and the USA. The call for abstracts received 39 submissions from a range of academic and humanitarian organizations such as LSHTM, American University of Beirut, Relief International, International Rescue Committee, Multiple Aid Program and other international and local NGOs. Some of the presentations were delivered through videoconferencing to ensure the inclusion of Syria-based researchers, as well as researchers based in neighbouring countries who might face travel and visa restrictions for presenting their research findings.

This 1-day conference included three keynote speakers: Dr Ayman Al Jundi from the Syrian British Medical Society, who has played a vital role in training medical personnel in Syria since the start of the conflict; Dr Munzer Khalil, Idlib Health Director, who presented virtually from Syria and Dr Fouad Mohammad Fouad from the American University of Beirut, on the informal nature of healthcare for Syrian refugees in Lebanon. Other presentations explored a number of research themes such as health research, medical education and training, health outcomes ranging from NCDs to infectious diseases through a conflict-specific lens, health needs of refugees and internally displaced populations, health system governance and adaption in the Syrian conflict and conflict-induced challenges and opportunities to re-building the health system and adapting programmes and activities to meet the needs of the population. To end the conference, a panel discussion highlighted the value of health research in conflict settings and how to encourage more research in such complex contexts.

## The Second International Syria Health Research Conference—30 October 2020

The organizing committee took the decision to make this conference an annual event to accelerate the considerable momentum generated after the first conference and to encourage further research and policy collaboration. Due to the coronavirus disease 2019 (COVID-19) pandemic, the conference was hosted virtually by Zoom. Although technically challenging, having an online conference allowed for a wider and more inclusive attendance, with around half of the presenters based outside the UK and around quarter based within Syria.

The second conference began with a welcoming address from Dr Maher Aref (UOSSM), based in Turkey, who shed light on the importance of health research and documentation from the perspective of health actors working on the ground in Syria. The main themes discussed during the second Syria research conference ranged from the impact of COVID-19 on health in Syria, health system governance and adaptation, NCDs to evaluations of clinical intervention and multidisciplinary research. The organizing committee received 26 abstracts of which 15 were accepted for oral presentations. The selection criteria for both conferences were based on judging the strength of methods, topic relevance and originality. Two keynote speeches were delivered: Dr Natasha Howard (SyRG-LSHTM/National University of Singapore), Principal Investigator of a Medical Research Council funded study on health system governance in Syria, and Professor Richard Sullivan (R4HSSS-KCL), Principal Investigator of a large National Institute of Health Research (NIHR) funded programme: Research for Health System Strengthening in northern Syria (R4HSSS). The day ended with a panel discussion on research for health system strengthening featuring experts representing several Syrian health organizations including the Idlib Health Directorate from opposition-controlled areas and the Health Department from the Autonomous-Administration-controlled areas of Syria. The panel stressed the need for more investment in research capacity strengthening for humanitarian and local actors in Syria, the importance of bottom-up and participatory approaches for the health research agenda, the key role of innovative research methodologies adapted for the Syrian context, and the significance of policy-relevant research in supporting the ongoing humanitarian efforts as well as generating new knowledge.

## Providing a platform and voice for Syrian researchers based inside Syria

A unique aspect of the annual conference is to strongly endorse equitable partnership between various academic, humanitarian and policy actors involved, as well as its promotion of the localization agenda. Agreed as part of the humanitarian reform process beginning around the World Humanitarian Summit in 2016, the localization agenda calls for greater inclusion of local actors in the humanitarian sector to address unequal power relations within the humanitarian system.^6^^,^^7^ This is manifested by the founding organizing committee consisting of Syrian researchers, co-hosted by academic and humanitarian entities. Moreover, the majority of submitted and accepted abstracts were received from Syria-based and diaspora Syrian researchers.

Both conferences attracted an extensive range of abstract submissions of research projects including 20% from Syrian researchers and research institutions in the first conference, and over 40% in the second conference. This was also reflected in the presentations selected on the day, whereby the majority of presentations were carried out by researchers from within Syria, neighbouring countries or Syrian diaspora elsewhere. The geographical distribution of the abstract submissions was a factor which further enriched the conference, as submissions were received from the various areas of control in Syria including Latakia, Damascus, Aleppo and Idleb. We also received submissions from Lebanon, Turkey, Jordan as well as numerous other countries globally including Canada, USA, Germany and the UK.

Both conferences demonstrated the need for strengthened research capacity inside Syria in relation to research design, methodology and dissemination as well as building a research culture on organizational and institutional level. More engagement with individual Syria-based researchers, as well as with local and international humanitarian actors is a step in the right direction. The involvement of local researchers will not only strengthen the capacity of individual researchers and their collaborating teams, but it will also increase the authenticity and relevance of the methodology, data and findings. Additionally, such involvement will support translating research findings into practice more effectively. Research findings tend to have more uptake by international and local actors involved in shaping policies and practices shall these actors be engaged in designing, implementing and disseminating these research. This inclusive approach has repeatedly demonstrated its ability as an effective tool in disadvantaged communities and humanitarian settings in both research and programmes.^8^^,^^9^ There is also a need for more focus on engaging and training junior and mid-career researchers to support their research careers and aspirations. Such engagement should consider the various forms of training, mentorship and coaching to meet the research capacity needs of junior and mid-career researchers.

## More equitable partnerships leading to improved research

The literature on global health partnerships highlights their role in supporting public health and health systems, developing education and training, and creating knowledge from high-quality research on key issues for the benefit of practitioners, policymakers and the public.^10–14^ Partnership work in Syria is an important step to realize the benefits of local knowledge and expertise, whilst supporting the implementation of broad health system goals by drawing on the strengths of international networks.

The vital importance of ensuring the development of equitable partnerships illustrates some of the prior criticisms of the partnership approach in other settings. In the context of research on Syrian refugees, there have been examples where engagement with local researchers were criticized for alienation, exploitation and disillusionment.^15^ Shared-decision making and resource distribution are key areas of focus in a strong collaboration, which when effectively delivered can be highly adaptable to local needs and unexpected crises.^13^^,^^14^ Developing this model of collaborative work in Syria has the potential to amplify dividends on offer by external research actors to support and strengthen health responses to local needs, whilst embedding the necessary skills and structure to rebuild the health system as it recovers from over a decade of war.

The 2019 and 2020 *Research for Health in the Syrian Conflict* conferences have clearly demonstrated that there is vast scope to conduct more health research inside Syria, the advantages of which are becoming more widely acknowledged by the international community. The benefit and impact of strengthening health research capacity is invaluable in developing and implementing healthcare policies that direct and adequately prioritize the required resources and funding that will in turn support improved health outcomes of individuals inside Syria.^16–18^

As proposed by the conferences, to achieve these research outcomes, equitable partnerships between organizations and individual researchers, both inside and outside of Syria, must form the basis of conducting high-quality and meaningful research. Ensuring the inclusion of local Syrian researchers is acutely crucial in the development of any viable partnership. Such partnerships can provide support in overcoming the numerous barriers of conducting research in precarious settings, for example ethical and security-related challenges.^19^ In the Syrian context, capacity strengthening in health research is urgently needed in its current phase of active conflict to inform, develop and implement strengthened and sustainable health systems in the post-conflict phase.

The institutions involved in organizing this conference have already taken practical steps to form such partnerships. Based on the R4HC-MENA project experience, the CHRG has partnered with UOSSM on multiple research grants applications. This has led to a successful grant application to the National Institute for Health Research in the UK for a health system strengthening project in Syria. The 4-year multimillion project is co-led by CHRG and UOSSM, and it has a wider network of partners including the SPHN, David Nott Foundation, SAMS and the Idleb Health Directorate, as well as other local and international partners. The project has an operational design that aims to study the experience of health systems in north west and north east Syria in relation to four elements of health system adaptation and strengthening: provision of health services, health education and medical training, health governance and financing and the use of digital solutions in health information system to inform future post conflict systems strengthening.^20^

Learning from the initial findings of these nascent networks and collaborations, we conclude that there is considerable global momentum from Syrian researchers, diaspora and policy makers to address health, security and development challenges posed by almost a decade of armed conflict and complex geopolitics that has resulted in different areas of political control. However, research funders have been so far reluctant to invest in large-scale research programmes in severely conflict-affected areas such as northern Syria. Large programmes such as the NIHR funded R4HSSS could change this and other funders will hopefully follow suit. Convincing funders to invest and engage more effectively is always going to be challenging and extremely competitive. However, equitable partnerships (at times founded by Syrian researchers themselves) with operational humanitarian organizations like UOSSM and academic organizations is a leading way forward to capitalize and accelerate the global momentum for conducting health research in armed conflict contexts.

## Authors’ contributions

The commentary framing, outlines, literature review, and initial drafting of the piece, multiple rounds of edits, and producing the final manuscript were carried out by A.E. G.B., K.M. and P.P. contributed to further literature review, additional content, initial drafting of specific sections and a round of edits. A.O., Y.D. and B.F. contributed to additional content, initial drafting of specific sections and a round of edits. All authors read, edited and approved the manuscript.

## Data Availability

Not applicable.

## Notes

**Abdulkarim Ekzayez,** MD MSc

**Amina Olabi,** MSc

**Yazan Douedari,** MSc

**Kristen Meagher,** Ms

**Gemma Bowsher,** MD MSc

**Bashar Farhat,** MD

**Preeti Patel,** PhD Prof.
